# Amphibians and reptiles of the state of Coahuila, Mexico, with comparison with adjoining states

**DOI:** 10.3897/zookeys.593.8484

**Published:** 2016-05-26

**Authors:** Julio A. Lemos-Espinal, Geoffrey R. Smith

**Affiliations:** 1 Laboratorio de Ecología-UBIPRO, FES Iztacala UNAM. Avenida los Barrios 1, Los Reyes Iztacala, Tlalnepantla, edo. de México, Mexico – 54090; 2 Department of Biology, Denison University, Granville, OH, USA 43023

**Keywords:** Biogeography, Checklist, Conservation Status, Herpetofauna, IUCN Red List

## Abstract

We compiled a checklist of the amphibians and reptiles of the state of Coahuila, Mexico. The list comprises 133 species (24 amphibians, 109 reptiles), representing 27 families (9 amphibians, 18 reptiles) and 65 genera (16 amphibians, 49 reptiles). Coahuila has a high richness of lizards in the genus *Sceloporus*. Coahuila has relatively few state endemics, but has several regional endemics. Overlap in the herpetofauna of Coahuila and bordering states is fairly extensive. Of the 132 species of native amphibians and reptiles, eight are listed as Vulnerable, six as Near Threatened, and six as Endangered in the IUCN Red List. In the SEMARNAT listing, 19 species are Subject to Special Protection, 26 are Threatened, and three are in Danger of Extinction. Coahuila is home to several species of conservation concern, especially lizards and turtles. Coahuila is an important state for the conservation of the native regional fauna.

## Introduction

**Figure 1.**
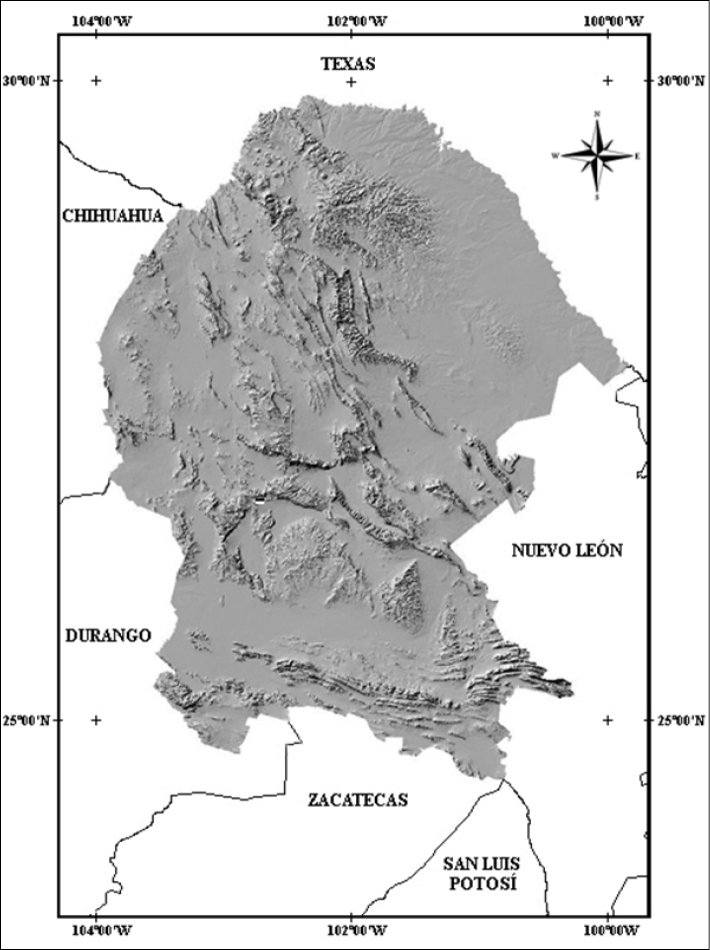
Topographical map of the state of Coahuila (INEGI 2001).

Coahuila is the third largest state of Mexico, encompassing 151,571 km2, between latitudes 24°32'S and 29°53'N and between longitudes 99°51'E and 103°58'W. It is bordered by the Rio Grande of Texas to the north, by the states of Durango, Zacatecas, and San Luis Potosí to the south, Chihuahua to the west, and Nuevo León to the east (Fig. 1). It represents 7.74% of the total area of Mexico.

Extensive sierras in the northern part of the state appear to form a single mountain mass, although they are actually composed of three ranges: Sierra El Carmen, the western third; Sierra El Burro, the eastern third; and Sierra de Santa Rosa, the southern third. The greatest altitude (2,120 m) is reached in the Sierra de Santa Rosa (28°18'N, 102°4'W). These sierras constitute about 40–50% of the northern part of the state; the rest of the northern part consists of plains whose average elevation is 1,000 m. In the extreme western part of the state, isolated, relatively small sierras, oriented north to south, arise abruptly from the arid/semiarid plains. The principal ones are Sierra Las Cruces, Sierra Mojada, Sierra El Pino and Sierra de Tlahualilo. The highest of these is Sierra Mojada (27°16'N, 103°42W), with a maximum altitude of 2,450 m. Around these mountains the plains, at an average altitude of 1,250 m, are dominated by areas of sand dunes. One set lies between Estación Sabaneta and an area east of Jaco (Chihuahua), a part of the Bolsón de Mapimí. Another is on the plains of Aguanaval east of the Sierra de Tlahualilo (Dunas Magnéticas), part of the Zona del Silencio. Still another is on the plains of the municipalities of Matamoros and Viesca, located in the extreme southwestern part of the area known as the Laguna de Mayrán. The extreme south central and southeastern parts of the state are characterized by a series of east-west crustal folds forming several sierras, notably the Sierra de Arteaga, Sierra La Concordia, and Sierra de Parras, contiguous to the east with the Sierra Madre Oriental. Cerro La Nopalera (25°8'N, 103°14'W), at 3,120 m, is the highest elevation in the area. Toward the southwest these ranges are continuous with those that form the southern limit of the Laguna de Mayrán. The eastern part of the state is mostly flat, broken by several isolated, low ranges extending N-S, notably the Sierra Pájaros Azules (27°0'N, 100°53'W), reaching an altitude of 1,930 m, and Sierra La Gloria. In the central part of the state is a small, low (~750 m) valley of 120 km^2^ surrounded by mountain ranges with altitudes of up to 2,500 m. For tens of thousands of years this valley was of strictly internal drainage, fed by waters from several arroyos, creating a wide variety of aquatic habitats, including streams, wells, lakes and marshes. Its isolation and antiquity led to a high degree of endemism there (McCoy 1984). At present this valley is known as the Cuatro Ciénegas Basin.

Much of Coahuila lies within the Chihuahuan Desert. The highlands in the extreme southeastern corner, including the Sierra de Arteaga, are an exception, and constitute the extreme northern end of the Sierra Madre Oriental. The vegetative cover of the state is made up of six types of vegetation (Chihuahuan Desert Scrub; Tamaulipan Thornscrub; Montane Forest; Sacatal Grassland; and Aquatic, Subaquatic and Riparian Vegetation) and 12 plant communities, that basically correspond to three floral provinces: The Mexican Plateau, the Coastal Plain of the Northeast and the Sierra Madre Oriental (Rzedowski 1978; Lemos-Espinal and Smith 2015b).

Lemos-Espinal and Smith (2015b) reviewed herpetological studies previously done in the state of Coahuila, with the majority of herpetological collections in Coahuila focused in the central part of the state (Bolsón de Cuatro Ciénegas), the southwestern part of the state (Laguna de Mayrán), and the extreme southeastern part of the state (Sierra de Arteaga). Other important regions of the state remain poorly studied, such as the extreme northwestern part of the state, due to lack of road access to these regions. However, in recent years, new highways has been constructed allowing access to previously unstudied areas, for example the highway from Múzquiz to Ojinaga, that traverses the northwestern part of Coahuila and connects this area with extreme northeastern Chihuahua. It is anticipated that this highway will increase herpetological studies of this region which is home of two important protected areas in Mexico: Área de Protección de Flora y Fauna Cañón de Santa Elena (Chihuahua) and Área de Protección de Flora y Fauna Maderas del Carmen (Coahuila).

Here, we report the list of amphibians and reptiles that have been recorded so far for the state of Coahuila. While checklists for Coahuila are available (e.g., Lemos-Espinal and Smith 2007, 2015b), we expand on these earlier efforts by also collecting and summarizing the conservation statuses of each documented species. We also compare the observed list to those available for the five adjoining states in the United States and Mexico for which recent checklists are available (Texas, Chihuahua, Durango, San Luis Potosí, and Nuevo León). Our goal is to place this checklist into a regional and conservation context not available in the previously published checklists.

## Methods

We compiled the list of amphibians and reptiles of the state of Coahuila from the following sources: (1) our own field work; (2) specimens from the Laboratorio de Ecología - UBIPRO (LEUBIPRO) collections; (3) databases from the Comisión Nacional para el Conocimiento y Uso de la Biodiversidad (National Commission for the Understanding and Use of Biodiversity; CONABIO), including the 22 collections listed in Appendix I; and (4) a thorough examination of the available literature on amphibians and reptiles in the state. Species were included in the checklist only if we were able to confirm the record, either by direct observation or through documented museum records or vouchers in the state. In addition, we recorded the conservation status of each species based on three sources: 1) the IUCN Red List, 2) Environmental Viability Scores from Wilson et al. (2013a,b), and 3) listing in SEMARNAT (2010).

Scientific names used in this publication are based on the taxonomic list published in Lemos-Espinal (2015). The arrangement of the amphibian names follows Frost (2015) and arrangement of the reptile names follows Uetz and Hošek (2015). State lists used to compare the species composition between Coahuila and the adjoining states were: Lemos-Espinal and Smith (2015a: Chihuahua); Valdez-Lares et al. (2013: Durango); Lemos-Espinal and Dixon (2013: San Luis Potosí); Lemos-Espinal and Cruz (2015: Nuevo León); Dixon (2015: Texas). We modified the list provided by Valdez-Lares et al. (2013) to be able to compare it with the list of the rest of the states. These modifications were the following: we regarded the population of *Barisia imbricata* (Wiegmann) as *Barisia ciliaris* (Smith); 2) we regarded *Sceloporus edbelli* Smith et al. as part of *Sceloporus consobrinus* Baird & Girard; 3) we regarded *Sceloporus lineolateralis* Smith as part of *Sceloporus jarrovii* Cope; and 4) we regarded *Aspidoscelis scalaris* (Baird & Girard) as part of *Aspidoscelis gularis* (Baird & Girard). For these states we also determine the number of overlapping species.

## Results

We documented a total of 132 native species: 24 amphibians (four salamanders, 20 anurans) and 108 reptiles (11 turtles, 49 lizards, 48 snakes) (Tables 1, 2). These represent 26 families: 9 of amphibians (two of salamanders and seven of frogs), and 17 of reptiles (four of turtles, seven of lizards and six of snakes), and 64 genera: 16 of amphibians (three of salamanders and 13 of frogs), and 48 of reptiles (six of turtles, 16 of lizards and 26 of snakes) (Tables 1, 2). Additionally, one introduced species, the Mediterranean House Gecko (*Hemidactylus turcicus*), was recorded.

**Table 1.** Checklist of amphibians and reptiles of Coahuila. We also provide the Habitat type (CD = Chihuahuan Desert, SM = Sierra Madre Oriental, TS = Tamaulipan Thornscrub), IUCN Status (DD = Data Deficient; LC = Least Concern, V = Vulnerable, NT = Neat Threatened; E = Endangered; CE = Critically Endangered) according to the IUCN Red List (The IUCN Red List of Threatened Species, Version 2014.2; www.iucnredlist.org; accessed 2 December 2015), Environmental Vulnerability Score (EVS; the higher the score the greater the vulnerability) from Wilson et al. (2013a,b), and conservation status in Mexico according to SEMARNAT (2010) (P = in danger of extinction, A = threatened; Pr = subject to special protection, NL – not listed). Source denotes whether the species was observed in the field by the authors (A), documented in the CONABIO data base and/or museum collections (C/M), or found in the literature (citation of source).

	Habitat Type	IUCN Status	EVS Score	SEMARNAT listing	Source
Class Amphibia					
Order Caudata					
Family Ambystomatidae					
*Ambystoma marvortium* Baird	CD	?	10	NL	Lemos-Espinal and Smith (2007)
Family Plethodontidae					
*Chiropterotriton priscus* Rabb	SM	?	16	Pr	C/M
*Pseudoeurycea galeanae* Taylor	SM	NT	18	A	C/M
*Pseudoeurycea scandens* Walker	SM	V	17	NL	C/M
Order Anura					
Family Bufonidae					
*Anaxyrus cognatus* (Say)	CD	LC	9	NL	A
*Anaxyrus debilis* (Girard)	CD	LC	7	Pr	A
*Anaxyrus punctatus* (Baird & Girard)	CD	LC	5	NL	A
*Anaxyrus speciosus* (Girard)	CD	LC	12	NL	A
*Anaxyrus woodhousii* (Girard)		LC	10	NL	A
*Incilius nebulifer* (Girard)		LC	6	NL	
*Rhinella marina* (Linnaeus)	CD	LC	3	NL	C/M
Family Craugastoridae					
*Craugastor augusti* (Dugès)	SM	LC	8	NL	C/M
Family Eleutherodactylidae					
*Eleutherodactylus guttilatus* (Cope)	SM	LC	11	NL	C/M
*Eleutherodactylus longipes* (Baird)	SM	V	15	NL	C/M
*Eleutherodactylus marnockii* (Cope)	CD	LC	?	NL	Lemos-Espinal and Smith (2007)
Family Hylidae					
*Acris crepitans* Baird	CD	LC	?	NL	
*Ecnomiohyla miotympanum* (Cope)	SM	NT	9	NL	Garza-Tobón and Lemos-Espinal (2013b)
*Hyla arenicolor* Cope	CD	LC	7	NL	A
*Smilisca baudinii* (Duméril & Bibron)	SM	LC	3	NL	Lemos-Espinal and Smith (2007)
Family Microhylidae					
*Gastrophryne olivacea* (Hallowell)	CD	LC	9	Pr	A
Family Ranidae					
*Lithobates berlandieri* (Baird)	CD	LC	7	Pr	C/M
*Lithobates catesbeianus* (Shaw)	CD, RIP	LC	10	NL	Garza-Tobón and Lemos-Espinal (2013b)
Family Scaphiopodidae					
*Scaphiopus couchii* Baird	CD	LC	3	NL	A
*Spea multiplicata* (Cope)	CD	LC	6	NL	A
Class Reptilia					
Order Testudines					
Family Emydidae					
*Pseudemys gorzugi* Ward	CD	NT	16	A	C/M
*Terrapene coahuila* Schmidt & Owens		E	19	A	C/M
*Trachemys gaigeae* (Hartweg)		V	18	NL	A
*Trachemys scripta* (Thusberg)	CD	LC	16	Pr	C/M
*Trachemys taylori* (Legler)	CD	E	19	NL	C/M
Family Kinosternidae					
*Kinosternon durangoense* Iverson	CD	DD	16	NL	A
*Kinosternon flavescens* (Agassiz)	CD	LC	12	NL	C/M
*Kinosternon hirtipes* (Wagler)		LC	10	Pr	C/M
Family Testudinae					
*Gopherus berlandieri* (Agassiz)	TS	LC	18	A	A
*Gopherus flavomarginatus* Legler	CD	V	19	P	A
Family Trionychidae					
*Apalone spinifera* (Le Sueur)	CD	LC	15	Pr	A
Order Squamata					
Suborder Lacertilia					
Family Anguidae					
*Barisia ciliaris* (Smith)	SM	?	15	NL	A
*Gerrhonotus infernalis* Baird	SM	LC	13	NL	A
*Gerrhonotus lugoi* McCoy	CD	LC	17	A	C/M
Family Crotaphytidae					
*Crotaphytus antiquus* Axtell & Webb	CD	E	16	NL	A
*Crotaphytus collaris* (Say)	CD	LC	13	A	A
*Crotaphytus reticulatus* Baird	TS	V	12	A	A
*Gambelia wislizenii* (Baird & Girard)	CD	LC	13	Pr	A
Family Eublepharidae					
*Coleonyx brevis* Stejneger	CD	LC	14	Pr	A
*Coleonyx reticulatus* Davis & Dixon	CD	LC	15	Pr	C/M
Family Gekkonidae					
*Hemidactylus turcicus* (Linnaeus)	CD	N/A	N/A	N/A	A
Family Phrynosomatidae					
*Cophosaurus texanus* Troschel	CD	LC	14	A	A
*Holbrookia approximans* Baird	CD	?	14	NL	A
*Holbrookia lacerata* Cope	CD, TS	NT	14	A	A
*Phrynosoma cornutum* (Harlan)	CD	LC	11	NL	A
*Phrynosoma modestum* Girard	CD	LC	12	NL	A
*Phrynosoma orbiculare* (Linnaeus)	SM	LC	12	A	A
*Sceloporus bimaculosus* Phelan & Brattstrom	CD	NL	?	NL	A
*Sceloporus cautus* Smith	CD	LC	15	A	C/M
*Sceloporus consobrinus* Baird & Girard	CD	?	?	NL	A
*Sceloporus couchii* Baird	CD	LC	15	NL	C/M
*Sceloporus cyanogenys* Cope	CD	?	16	NL	A
*Sceloporus cyanostictus* Axtell & Axtell	CD	E	13	NL	A
*Sceloporus goldmani* Smith	CD	E	15	NL	C/M
*Sceloporus grammicus* Wiegmann	SM, TS	LC	9	Pr	A
*Sceloporus maculosus* Smith	CD	V	16	Pr	A
*Sceloporus merriami* Stejneger	CD	LC	13	NL	A
*Sceloporus minor* Cope	SM	LC	14	NL	A
*Sceloporus oberon* Smith & Brown	SM	V	14	NL	A
*Sceloporus olivaceus* Smith	TS	LC	13	NL	A
*Sceloporus ornatus* Baird	CD	NT	16	A	C/M
*Sceloporus parvus* Smith	CD	LC	15	NL	A
*Sceloporus poinsettii* Baird & Girard	CD	LC	12	NL	A
*Sceloporus samcolemani* Smith & Hall	Grassland CD	LC	15	NL	C/M
*Sceloporus spinosus* Wiegmann	CD	LC	12	NL	C/M
*Sceloporus variabilis* Wiegmann	SM	LC	5	NL	A
*Uma exsul* Schmidt & Bogert	CD	E	16	P	A
*Uma paraphygas* Williams, Chrapliwy & Smith	CD	NT	17	P	A
*Urosaurus ornatus* (Baird & Girard)	CD	LC	10	NL	A
*Uta stansburiana* Baird & Girard	CD	LC	11	A	A
Family Scincidae					
*Plestiodon dicei* (Ruthven & Gaige)	SM	LC	7	NL	A
*Plestiodon obsoletus* (Baird & Girard)	CD	LC	11	NL	A
*Plestiodon tetragrammus* Baird	CD	LC	12	NL	A
*Scincella kikaapoa* (García-Vázquez, Canseco-Márquez, & Nieto Montes de Oca)	CD	NL	17	NL	García-Vázquez et al. (2010)
*Scincella lateralis* (Say)		LC	13	Pr	C/M
*Scincella silvicola* (Taylor)	SM	LC	12	A	García-Vázquez et al. (2005)
Family Teiidae					
*Aspidoscelis gularis* (Baird & Girard)	CD	LC	9	NL	A
*Aspidoscelis inornata* (Baird)	CD	LC	14	NL	A
*Aspidoscelis marmorata* (Baird & Girard)	CD	?	14	NL	A
*Aspidoscelis tesselata* (Say)	CD, RIP	LC	14	NL	A
Family Xantusidae					
*Xantusia extorris* Webb	CD	LC	15	NL	Castañeda-Gaytan et al. (2013)
Order Squamata					
Suborder Serpentes					
Family Colubridae					
*Arizona elegans* Kennicott	CD	LC	5	NL	A
*Bogertophis subocularis* (Brown)	CD	LC	14	NL	A
*Coluber constrictor* Linnaeus	Grassland in CD & SM	LC	10	A	C/M
*Drymarchon melanurus* (Duméril, Bibron & Duméril)	SM	LC	6	NL	A
*Gyalopion canum* Cope	CD	LC	9	NL	C/M
*Lampropeltis alterna* (Brown)	CD	LC	14	A	A
*Lampropeltis getula* (Blainville)	CD	LC	?	A	A
*Lampropeltis mexicana* (Garman)	SM	LC	15	A	A
*Lampropeltis triangulum* (Lacèpéde)	CD	?	7	A	C/M
*Masticophis flagellum* (Shaw)	CD	LC	8	A	A
*Masticophis schotti* Baird & Girard	CD, TS	LC	13	NL	A
*Masticophis taeniatus* (Hallowell)	CD	LC	10	NL	A
*Opheodrys aestivus* (Linneaus)	SM	LC	13	NL	C/M
*Pantherophis bairdi* (Yarrow)	CD	LC	15	NL	C/M
*Pantherophis emoryi* (Baird & Girard)	CD	LC	13	NL	A
*Pituophis catenifer* Blainville	CD	LC	9	NL	A
*Pituophis deppei* (Duméril)	SM	LC	14	A	C/M
*Rhinocheilus lecontei* Baird & Girard	CD	LC	8	NL	A
*Salvadora grahamiae* Baird & Girard	CD	LC	10	NL	A
*Sonora semiannulata* Baird & Girard	CD	LC	5	NL	A
*Tantilla atriceps* (Günther)	CD	LC	11	A	C/M
*Tantilla gracilis* Baird & Girard		LC	13	A	C/M
*Tantilla hobartsmithi* Taylor	CD	LC	11	NL	A
*Tantilla nigriceps* Kennicott	CD	LC	11	NL	A
*Tantilla wilcoxi* Stejneger	CD	LC	10	NL	A
Family Dipsadidae					
*Diadophis punctatus* (Linnaeus)	SM	LC	4	NL	A
*Heterodon kennerlyi* Kennicott	CD	?	11	Pr	A
*Hypsiglena jani* (Dugès)	CD	?	6	NL Pr?	A
*Leptodeira septentrionalis* (Kennicott)	SM	?	8	NL	C/M
Family Elapidae					
*Micrurus tener* Baird & Girard	CD	LC	11	NL	A
Family Leptotyphlopidae					
*Rena dissecta* (Cope)		LC	11	NL	C/M
*Rena dulcis* Baird & Girard	CD	LC	13	NL	C/M
*Rena segrega* (Klauber)		NL	?	NL	C/M
Family Natricidae					
*Nerodia erythrogaster* (Forster)	CD	LC	11	A	A
*Nerodia rhombifer* (Hallowell)	CD	LC	10	NL	A
*Storeria hidalgoensis* Taylor	SM	V	13	NL	C/M
*Thamnophis cyrtopsis* Kennicott)	CD	LC	7	A	A
*Thamnophis exsul* (Baird & Girard)	SM	LC	16	NL	C/M
*Thamnophis marcianus* (Baird & Girard)	CD	LC	10	A	A
*Thamnophis proximus* (Say)	SM	LC	7	A	A
Family Viperidae					
*Agkistrodon contortrix* (Linnaeus)	CD	LC	14	NL	C/M
*Crotalus atrox* Baird & Girard	CD	LC	9	Pr	A
*Crotalus lepidus* (Kennicott)	CD	LC	12	Pr	A
*Crotalus molossus* Baird & Girard	CD	LC	8	Pr	A
*Crotalus pricei* Van Denburgh	SM	LC	14	Pr	A
*Crotalus scutulatus* (Kennicott)	CD	LC	11	Pr	A
*Crotalus viridis* (Rafinesque)	CD	LC	12	Pr	C/M
*Sistrurus catenatus* (Rafinesque)	CD	LC	13	Pr	C/M

**Table 2.** Summary of species present in Coahuila by Family, Order or Suborder, and Class. Status summary indicates the number of species found in each IUCN conservation status in the Order DD, LC, V, NT, E, CE (see Table 1 for abbreviations; in some cases species have not been assigned a status by the IUCN and therefore these may not add up to the total number of species in a taxon). Mean EVS is the mean Environmental Vulnerability Score, scores ≥ 14 are considered high vulnerability (Wilson et al. 2013a,b) and conservation status in Mexico according to SEMARNAT (2010) in the Order NL, Pr, A, P (see Table 1 for abbreviations).

Class	Order/ Suborder	Family	Genera	Species	Status Summary	Mean EVS	SEMARNAT
Amphibia	Caudata		**3**	**4**	**0,1,1,2,0,0**	15.25	**2,1,1,0**
		Ambystomatidae	1	1	0,1,0,0,0,0	10	1,0,0,0
		Plethodontidae	2	3	0,0,1,2,0,0	17	1,1,1,0
	Anura		**13**	**20**	**0,18,1,0,0,0**	**7.78**	**17,3,0,0**
		Bufonidae	3	7	0,7,0,0,0,0	7.43	6,1,0,0
		Craugastoridae	1	1	0,1,0,0,0,0	8	1,0,0,0
		Eleutherodactylidae	1	3	0,2,1,0,0,0	13	3,0,0,0
		Hylidae	4	4	0,3,0,0,0,0	6.33	4,0,0,0
		Microhylidae	1	1	0,1,0,0,0,0	9	0,1,0,0
		Ranidae	1	2	0,2,0,0,0,0	8.5	1,1,0,0
		Scaphiopodidae	2	2	0,2,0,0,0,0	4.5	2,0,0,0
	**Subtotal**		**16**	**24**	**0,19,2,2,0,0**	9.14	**19,4,1,0**
Reptilia							
	Testudines		**6**	**11**	**1,5,2,1,2,0**	**16.2**	**4,3,3,1**
		Emydidae	3	5	0,1,1,1,2,0	17.6	2,1,2,0
		Kinosternidae	1	3	1,2,0,0,0,0	12.67	2,1,0,0
		Testudinae	1	2	0,1,1,0,0,0	18.5	0,0,1,1
		Trionychidae	1	1	0,1,0,0,0,0	15	0,1,0,0
	Squamata						
	Lacertilia		**17**	**50**	**0,30,3,3,4,0**	**13.0**	**34,4,9,2**
		Anguidae	2	3	0,2,0,0,0,0	15	2,0,1,0
		Crotaphytidae	2	4	0,2,1,0,1,0	13.5	1,1,2,0
		Eublepharidae	1	2	0,2,0,0,0,0	14.5	2,0,0,0
		Gekkonidae	1	1	-		-
		Phrynosomatidae	7	29	0,16,2,3,3,0	13.3	20,2,5,2
		Scincidae	2	6	0,4,0,0,0,0	12	4,1,1,0
		Teiidae	1	4	0,3,0,0,0,0	12.75	4,0,0,0
		Xantusidae	1	1	0,1,0,0,0,0	15	1,0,0,0
	Serpentes		**26**	**48**	**0,42,1,0,0,0**	**10.5**	**27,8,13,0**
		Colubridae	14	25	0,24,0,0,0,0	10.6	16,0,9,0
		Dipsadidae	4	4	0,1,0,0,0,0	7.25	3,1,0,0
		Elapidae	1	1	0,1,0,0,0,0	11	1,0,0,0
		Leptotyphlopidae	1	3	0,2,0,0,0,0	12	3,0,0,0
		Natricidae	3	7	0,6,1,0,0,0	10.6	3,0,4,0
		Viperidae	3	8	0,8,0,0,0,0	11.6	1,7,0,0
	**Subtotal**		**49**	**109**	**1,77,6,4,6,0**	12.3	**65,15,25,3**
TOTAL			**65**	**133**	**1,96,8,6,6,0**		**84,19,26,3**

The difficult access to large and important parts of the state assure us that the number of native amphibian and reptile species that inhabits Coahuila is larger than the one we are reporting here. Species such as the Texas Salamander (*Eurycea neotenes* Bishop & Wright) and the Plains Spadefoot (*Spea bombifrons* [Cope]) likely inhabit extreme northern Coahuila. Dixon (2000) indicated the occurrence of these species at several localities in Texas adjacent to the extreme northern border of the state. The Ornate Box Turtle *Terrapene ornata* (Agassiz) very likely inhabits the Chihuahuan Desert of Coahuila, although as yet there are no records of this species in the state. According to Lemos-Espinal and Smith (2007, 2015b) species such as the Common Lesser Earless Lizard (*Holbrookia maculata* Girard), the Hernández Short-horned Lizard (*Phrynosoma hernandesi* Girard), and the Chihuahuan Spotted Whiptail (*Aspidoscelis exsanguis* [Lowe]), possibly inhabit extreme northwestern Coahuila. The Pigmy Alligator Lizard (*Gerrhonotus parvus* [Knight & Scudday]) may occur in the pine forests of the Sierra de Arteaga, and the Eastern Spiny Lizard (*Sceloporus spinosus* Weigmann) may occur in the semiarid region of the extreme southeastern part of the state. The Green Anole (*Anolis carolinensis* [Voigt]), the Laredo Striped Whiptail (*Aspidoscelis laredoensis* [McKinney et al.]), and the Six-lined Racerunner (*Aspidoscelis sexlineata* [Linnaeus]) probably occur in extreme northeastern Coahuila adjacent to Texas. The Torquate Lizard (*Sceloporus torquatus* Wiegmann) and the Bolson Night Lizard (*Xantusia bolsonae* Webb) likely occur in extreme southwestern Coahuila. Lemos-Espinal and Smith (2007, 2015b) also suggested that several species of snakes not recorded for Coahuila may inhabit the state, including Taylor´s Cantil (*Agkistrodon taylori* Burger & Robertson), the Tamaulipan Hook-nosed Snake (*Ficimia streckeri* Taylor), and the Red Black-headed Snake (*Tantilla rubra* Cope) in the southeastern portion of the state; the Tampico Threadsnake (*Rena myopica* [Garman]) and the Nuevo León Graceful Brownsnake (*Rhadinaea montana* Smith) in the extreme eastern portion; Dekay´s Brownsnake (*Storeria dekayi* [Holbrook]) and the Trans-Pecos Black-headed Snake (*Tantilla cucullata* Minton) in the extreme northeastern part; and the Big Bend Patch-nosed Snake (*Salvadora deserticola* Schmidt) and Texas Lyresnake (*Trimorphodon vilkinsonii* Cope) in the extreme northwestern part. Rossman et al. (1996) indicated the presence of the Mexican Gartersnake (*Thamnophis eques* (Reuss)), the Mexican Black-bellied Gartersnake (*Thamnophis melanogaster* [Peters]), and the Madrean Narrow-headed Gartersnake (*Thamnophis unilabialis* Tanner) in extreme southwestern Coahuila; however, no records for these species exist for Coahuila and we did not include them in the species list for this state.

**Figure 2.**
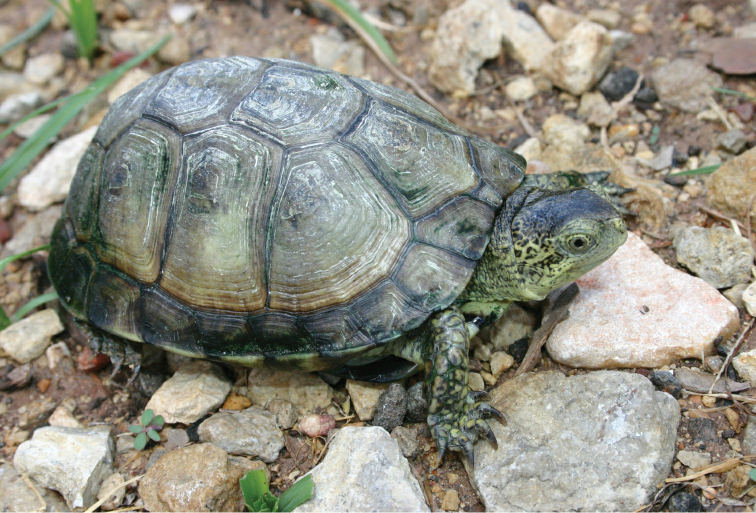
*Terrapene coahuila*. Cuatro Ciénegas, Coahuila. Species endemic to Coahuila. Photo courtesy of Michael Price.

**Figure 3.**
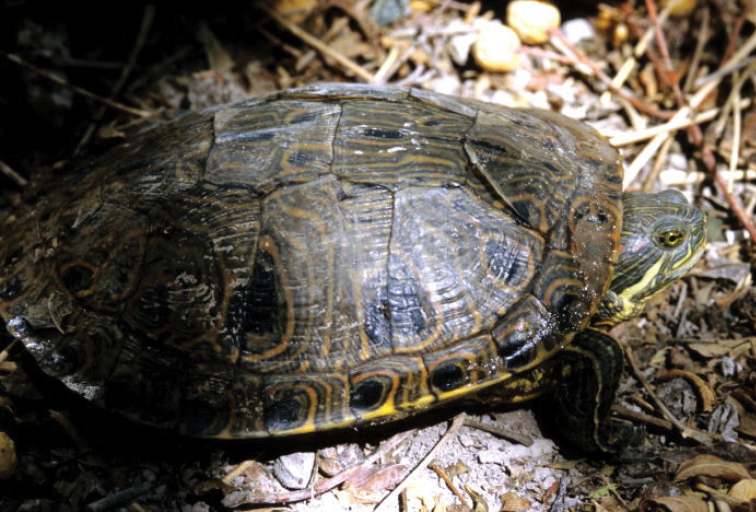
*Trachemys taylori*. Cuatro Ciénegas, Coahuila. Species endemic to Coahuila. Photo courtesy of Peter Heimes.

**Figure 4.**
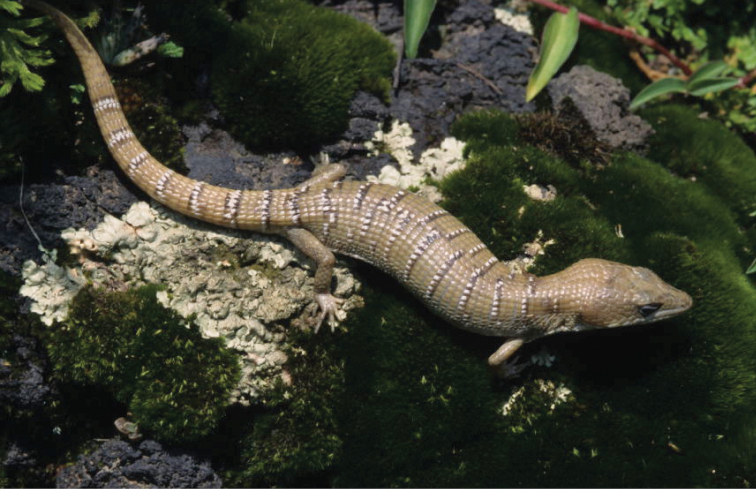
*Gerrhonotus lugoi*. Cuatro Ciénegas, Coahuila. Species endemic to Coahuila. Photo courtesy of Peter Heimes.

**Figure 5.**
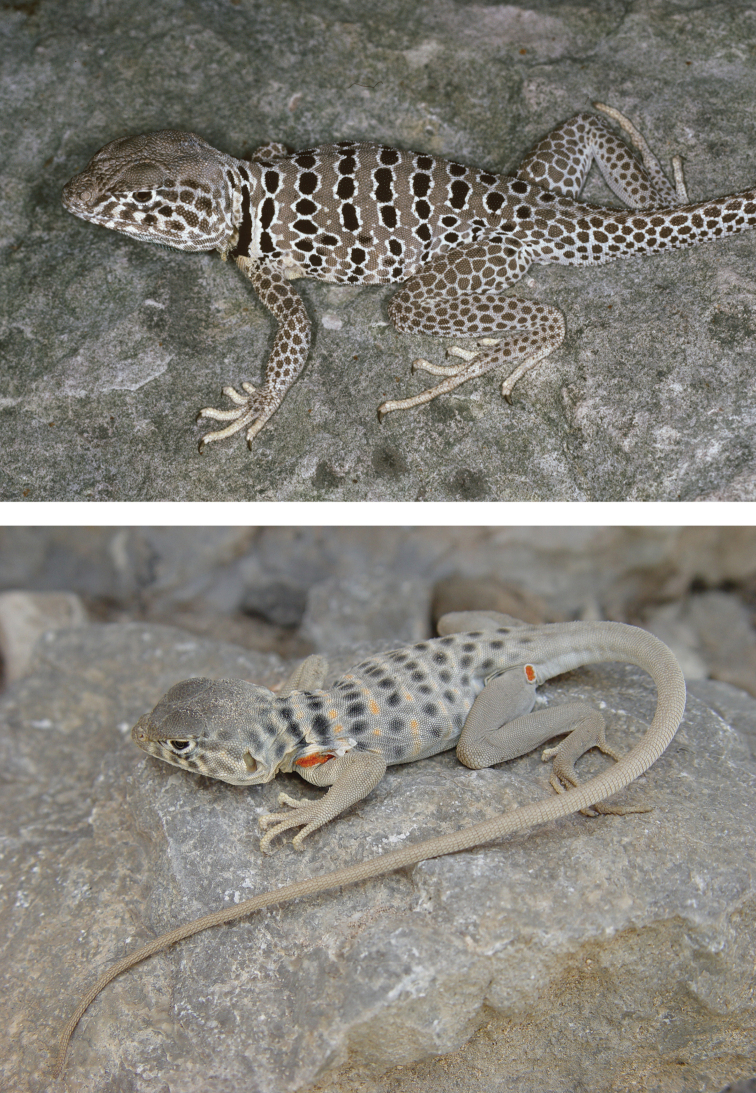
*Crotaphytus antiquus* (top: male; bottom: female). Sierra de San Lorenzo, Coahuila. Species endemic to Coahuila. Photos courtesy of Jimmy McGuire (male) and Tim Burkhardt (female).

**Figure 6.**
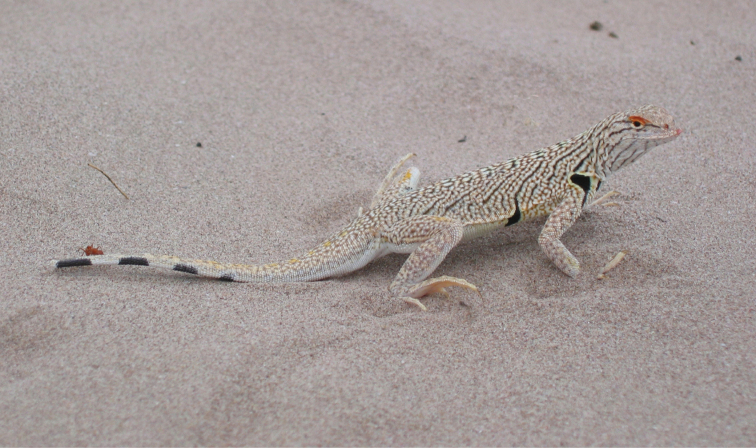
*Uma exsul* (male). Dunas de Bilbao, Viesca, Coahuila. Species endemic to Coahuila. Photo by Julio Lemos-Espinal.

Thirty five of the 132 species of amphibians and reptiles that inhabit Coahuila are endemic to Mexico, 20 of them are limited to areas of the Chihuahua Desert, including six endemic to Coahuila: *Terrapene coahuila* (Fig. 2), *Trachemys taylori* (Fig. 3), *Gerrhonotus lugoi* (Fig. 4), *Crotaphytus antiquus* (Fig. 5), *Uma exsul* (Fig. 6), and *Scincella kikaapoa*. Three of these six are limited to the Cuatro Ciénegas Bolson (*Terrapene coahuila*, *Gerrhonotus lugoi*, and *Scincella kikaapoa*), with one more, *Trachemys taylori* limited to the Cuatro Ciénegas Bolson and a small area around it. The other two Coahuila endemics, *Crotaphytus antiquus* and *Uma exsul*, are endemic to southwestern Coahuila. Four more species are limited to scattered regions of northern Mexico: *Sceloporus couchi* to the northern Sierras of Coahuila and central western Nuevo León; *Sceloporus goldmani* to a small area in southeastern Coahuila, adjacent Nuevo León, and northeastern San Luis Potosí; *Sceloporus maculosus* to the drainage of the Río Nazas in Durango and Coahuila; and *Xantusia extorris* to a small area in western Durango and southwestern Coahuila. Four more species are limited to the Mexican Plateau (*Sceloporus cautus*, *Sceloporus samcolemani*, and *Lampropeltis mexicana*) and central Mexico (*Sceloporus spinosus*). Another three species are limited to the small area of the Bolsón de Mapimí of southeastern Chihuahua, western Coahuila, and northeastern Durango (*Kinosternon durangoense*, *Gopherus flavomarginatus*, and *Uma paraphygas*). Two more species (*Sceloporus cyanostictus* and *Sceloporus ornatus*) are limited to Coahuila and extreme western Nuevo León. The last of these 35 endemic species (*Holbrookia approximans*) is limited to the Chihuahuan Desert of Mexico; however, it is highly likely that it occurs in adjacent parts of the United States. The remaining 15 endemic species are limited in eastern Mexico to the mountains and foothills of the Sierra Madre Oriental (*Chiropterotriton priscus*, *Pseudoeurycea galeanae*, *Pseudoeurycea scandens*, *Eleutherodactylus longipes*, *Ecnomiohyla miotympanum*, *Barisia ciliaris*, *Phrynosoma orbiculare*, *Sceloporus minor*, *Sceloporus oberon*, *Sceloporus parvus*, *Plestiodon dicei*, *Scincella silvicola*, *Pituophis deppei*, *Storeria hidalgoensis*, and *Thamnophis exsul*). These species enter Coahuila only in the southeastern corner of the state.

The remaining 97 of the 132 native species of amphibians and reptiles in Coahuila are not endemic, and all of them are shared with the United States; most of these shared species (95% = 93/98) occur in the Chihuahuan Desert and extend their ranges southward from the Great Plains of the United States to the southern tip of the Chihuahua Desert in the Mexican states of San Luis Potosí or Querétaro. Only four of these shared species are characteristic of the American tropics and subtropics (*Rhinella marina*, *Smilisca baudinii*, *Drymarchon melanurus*, and *Leptodeira septentrionalis*). *Rhinella marina* has been recorded in the lowlands of central Coahuila, in the semiarid Cuatro Ciénegas Bolson, whereas the other three occur in the lowlands of northeastern Coahuila and the western foothills of the Sierra Madre Oriental. All four of the species with tropical affinities enter the United States only in the southern part of Texas.

When comparing Coahuila to its surrounding states (Chihuahua, Durango, San Luis Potosí, Nuevo León, and Texas) we found that the total number of native species for these six states together is 451: 122 amphibians (38 salamanders and 85 anurans), and 323 reptiles (two crocodilians, 43 turtles, 120 lizards, and 164 snakes) (see Tables 3, 4). These represent 45 families: 15 of amphibians (six of salamanders and nine of frogs), and 30 of reptiles (one of crocodiles, eight of turtles, 14 of lizards, and seven of snakes), and 143 genera: 34 of amphibians (11 of salamanders and 23 of frogs), and 109 of reptiles (two of crocodilians, 19 of turtles, 29 of lizards, and 59 of snakes). Additionally, we found that there are 11 introduced species that maintain reproductive populations in one or more of these six states. One of these 11 introduced species, the Greenhouse Frog (*Eleutherodactylus planirostris* [Cope]), occurs naturally in Cuba and the West Indies and has been introduced to Texas; another one, the Brown Anole (*Anolis sagrei* Duméril & Bibron), occurs in Cuba, The Bahamas, the Yucatán Peninsula, and the northern part of Central America and has also been introduced to Texas. Another introduced species, the Florida Red-bellied Cooter (*Pseudemys nelsoni* Carr) is native to Florida and has been introduced to Texas. Another six non-native species, five of them belonging to the Family Gekkonidae, are native of Asia, Africa, and /or the Indo-Australian Archipielago: the Rough-tailed Gecko (*Mediodactylus scabrum* [Heyden]) introduced to Texas, the Stump-toed Gecko (*Gehyra mutilata* [Wiegmann]) introduced to San Luis Potosí, the Common House Gecko (*Hemidactylus frenatus* Schlegel) introduced to San Luis Potosí and Texas, the Indo-Pacific House Gecko (*Hemidactylus garnoti* Duméril & Bibron) introduced to Texas, the Mediterranean House Gecko (*Hemidactylus turcicus*) introduced to all six states, and the last species, belonging to the Family Typhlopidae, the Brahminy Blindsnake (*Indotyphlops braminus* [Daudin]) introduced to Durango, Nuevo León, and Texas. Two other species occur naturally in one or more of these six states but have been introduced to at least to one of these states where it does not occur naturally: the American Bullfrog (*Lithobates catesbeianus* [Shaw]) which ranges in southeastern Canada, central and eastern United States and eastern Mexico and has been introduced to Chihuahua, Durango, and San Luis Potosí, and the Mexican Spiny-tailed Iguana (*Ctenosaura pectinata* [Wiegmann]) which ranges in western Mexico and has been introduced to Texas.

**Table 3.** Total number of native amphibian and reptile species in each state arranged according to taxonomic Family (COH = Coahuila, CHI = Chihuahua, SLP = San Luis Potosí, DUR = Durango, NL = Nuevo León, TX = Texas).

	REGION	COH	CHI	SLP	DUR	NL	TX
CLASS AMPHIBIA							
Order CAUDATA							
Ambystomatidae	9	1	3	1	3	1	6
Amphiumidae	1	-	-	-	-	-	1
Plethodontidae	23	3	1	4	-	2	16
Proteidae	1	-	-	-	-	-	1
Salamandridae	2	-	-	1	-	-	2
Sirenidae	2	-	-	-	-	-	2
Order ANURA							
Bufonidae	16	7	10	6	9	6	10
Craugastoridae	6	1	2	3	4	1	1
Eleutherodactylidae	12	3	2	6	3	3	3
Hylidae	22	4	5	9	5	2	10
Leptodactylidae	2	-	-	2	-	1	1
Microhylidae	4	1	3	2	1	2	3
Ranidae	18	2	8	4	5	1	8
Rhinophrynidae	1	-	-	1	-	1	1
Scaphiopodidae	4	2	3	2	2	3	4
CLASS REPTILIA							
Order CROCODYLIA							
Crocodylidae	2	-	-	1	-	-	1
Order TESTUDINES							
Chelonidae	4	-	-	-	-	-	4
Chelydridae	2	-	-	-	-	-	2
Dermochelyidae	1	-	-	-	-	-	1
Emydidae	20	5	4	2	1	2	15
Geoemydidae	1	-	1	-	-	-	-
Kinosternidae	10	3	5	4	3	2	5
Testudinidae	3	2	2	-	1	1	1
Trionychidae	2	1	1	1	-	1	2
Order SQUAMATA							
Suborder LACERTILIA							
Anguidae	12	3	4	5	4	3	2
Corytophanidae	2	-	-	2	-	-	-
Crotaphytidae	4	4	2	1	2	2	3
Dactyloidae	5	-	1	2	1	-	1
Dibamidae	1	-	-	1	-	-	-
Eublepharidae	4	2	1	1	2	1	2
Helodermatidae	1	-	1	-	1	-	-
Iguanidae	3	-	1	1	1	-	-
Phrynosomatidae	51	29	24	19	26	27	19
Phyllodactylidae	1	-	1	-	1	-	-
Scincidae	16	6	7	5	5	4	8
Teiidae	13	4	8	3	4	3	10
Xantusidae	6	1	-	4	2	1	-
Xenosauridae	1	-	-	1	-	-	-
Suborder SERPENTES							
Boidae	1	-	1	1	1	-	-
Colubridae	65	25	35	36	31	31	33
Dipsadidae	33	4	10	19	7	8	9
Elapidae	3	1	2	1	-	1	1
Leptotyphlopidae	5	3	3	3	1	2	3
Natricidae	36	7	12	12	12	10	19
Viperidae	20	8	10	10	7	8	10
TOTAL	**451**	**132**	**172**	**177**	**145**	**130**	**220**

**Table 4.** Total number of native amphibian and reptile species in each state arranged according to taxonomic Order/Suborder (abbreviations as in Table 3).

	COH	CHI	SLP	DUR	NL	TX
Order/Suborder						
Caudata	4	4	6	3	3	28
Anura	20	33	35	29	20	41
Crocodilia			1			1
Testudina	11	13	7	5	6	30
Squamata/Lacertilia	49	50	45	49	41	45
Squamata/Serpentes	48	73	82	59	60	75
TOTAL	132	173	176	145	130	220

Coahuila shares the most species with Nuevo León and Texas, and shares fewer species with Chihuahua, Durango, and San Luis Potosí (Table 5). The other states share several species with each other. The two states that share the highest number of species are Chihuahua and Durango with 108 species shared, followed by Coahuila and Nuevo León with 102 shared species. The lowest numbers of shared species are found between Chihuahua and San Luis Potosí (61), Durango and Texas (61), Chihuahua and Nuevo León (65), and Durango and Nuevo León (65).

**Table 5.** Number of shared species between the six analyzed states (abbreviations as in Table 3).

	COH	CHI	DUR	SLP	NL	TX
COH	-	75	72	74	102	94
CHI		-	108	61	65	81
DUR			-	67	65	61
SLP				-	93	66
NL					-	85
TX						-

Thirty seven species are present in all the six states that we compared: *Anaxyrus cognatus*, *Anaxyrus debilis*, *Anaxyrus punctatus*, *Rhinella marina*, *Craugastor augusti*, *Smilisca baudinii*, *Gastrophryne olivacea*, *Lithobates berlandieri*, *Scaphiopus couchi*, *Spea multiplicata*, *Crotaphytus collaris*, *Cophosaurus texanus*, *Phrynosoma cornutum*, *Phrynosoma modestum*, *Sceloporus consobrinus*, *Sceloporus poinsettii*, *Plestiodon obsoletus*, *Aspidoscelis gularis*, *Aspidoscelis inornata*, *Arizona elegans*, *Drymarchon melanurus*, *Gyalopion canum*, *Lampropeltis getula*, *Masticophis flagellum*, *Pantherophis emoryi*, *Pituophis catenifer*, *Rhinocheilus lecontei*, *Salvadora grahamiae*, *Diadophis punctatus*, *Heterodon kennerlyi*, *Hypsiglena jani*, *Thamnophis cyrtopsis*, *Thamnophis marcianus*, *Crotalus atrox*, *Crotalus lepidus*, *Crotalus molossus*, and *Crotalus scutulatus*.

Twenty-three species are present in all but one of the six states that we compared. There are 10 species that are absent only in San Luis Potosí: *Ambystoma mavortium*, *Coleonyx brevis*, *Sceloporus merriami*, *Uta stansburiana*, *Aspidoscelis marmorata*, *Bogertophis subocularis*, *Masticophis taeniatus*, *Sonora semiannulata*, *Tantilla nigriceps*, and *Nerodia erythrogaster*. The main distribution of most of these species involves the North American deserts and have their southernmost distributions slightly north of San Luis Potosí. Another six of these 23 species are absent in Texas, four of them are species endemic to Mexico: *Barisia ciliaris*, *Holbrookia approximans*, *Phrynosoma orbiculare*, and *Pituophis deppei*, and two more are species that are distributed far to the south or west of Texas: *Tantilla wilcoxi*, and *Crotalus pricei*. Three more species are absent in Chihuahua: *Gerrhonotus infernalis*, *Sceloporus grammicus*, and *Tantilla atriceps*. Another three are absent from Durango: *Apalone spinifera*, *Plestiodon tetragrammus*, and *Lampropeltis triangulum*, and one more is absent in Nuevo León: *Kinosternon hirtipes* (Wagler). Texas is the only state with a marine coast in the Gulf of Mexico and thus is the only state with sea turtles: Loggerhead Sea Turtle (*Caretta caretta* [Linnaeus]), Green Sea Turtle (*Chelonia mydas* [Linnaeus]), Hawksbill Sea Turtle (*Eretomochelys imbricata* [Linnaeus]), Kemp’s Ridley Sea Turtle (*Lepidochelys kempii* [Garman]), and Leatherback Sea Turtle (*Dermochelys coriacea* [Vandelli]).

On the other hand, the region hosts 35 endemic species, 20 of them endemic to Texas: Salado Salamander (*Eurycea chisholmensis* Chippindale et al.), Cascade Caverns Salamander (*Eurycea latitans* Smith & Potter), San Marcos Salamander (*Eurycea nana* Bishop), Georgetown Salamander (*Eurycea naufragia* Chippindale et al.), Texas Salamander (*Eurycea neotenes* Bishop & Wright), Fern Bank Salamander (*Eurycea pterophila* Burger et al.), Texas Blind Salamander (*Eurycea rathbuni* [Stejneger]), Blanco Blind Salamander (*Eurycea robusta* [Potter & Sweet]), Barton Springs Salamander (*Eurycea sosorum* Chippindale et al.), Jollyville Plateau Salamander (*Eurycea tonkawae* Chippindale et al.), Comal Blind Salamander (*Eurycea tridentifera* Mitchell & Reddell), Valdina Farms Salamander (*Eurycea troglodytes* Baker), Austin Blind Salamander (*Eurycea waterlooensis* Hillis et al.), and Western Slimy Salamander (*Plethodon albagula* Grobman), Houston Toad (*Anaxyrus houstonensis* [Sanders]), Cagle’s Map Turtle (*Graptemys caglei* Haynes & McKown), Texas Map Turtle (*Graptemys versa* Stejneger), Texas Cooter (*Pseudemys texana* Bauer), Trans-Pecos Black-headed Snake (*Tantilla cucullata* Minton), and Harter’s Watersnake (*Nerodia harteri* [Trapido]); six more to Coahuila: Coahuilan Box Turtle (*Terrapene coahuila*), Cuatrociénegas Slider (*Trachemys taylori*), Lugo’s Alligator Lizard (*Gerrhonotus lugoi*), Venerable Collared Lizard (*Crotaphytus antiquus*), Fringe-toed Sand Lizard (*Uma exsul*), and Cuatrociénegas Little Skink (*Scincella kikaapoa*); three more to Chihuahua: Lemos-Espinal’s Leopard Frog (*Lithobates lemosespinali* [Smith & Chiszar]), Chihuahuan Alligator Lizard (*Barisia levicollis* Stejneger), and Chihuahuan Skink (*Plestiodon multilineatus* [Tanner]); another three to Durango: Bolson Night Lizard (*Xantusia bolsonae* Webb), Fox’s Mountain Meadow Snake (*Adelophis foxi* Rossman & Blaney), and Durango Spotted Garthersnake (*Thamnophis nigronuchalis* Thompson); two to Nuevo León: Pigmy Alligator Lizard (*Gerrhonotus parvus* [Knight & Scudday]) and Nuevo León Graceful Brown Snake (*Rhadinaea montana* Smith); and only one to San Luis Potosí: Newman’s Knob-scaled Lizard (*Xenosaurus newmanorum* Taylor).

## Discussion

Like many other states in Mexico, Coahuila has a rich herpetofauna, but especially a rich reptile fauna. In particular, Coahuila has a high diversity of lizards in the genus *Sceloporus* (19 species). The richness of reptiles is consistent with the importance of desert habitats in Coahuila. Despite its richness in reptiles and amphibians, Coahuila has a relatively small number of endemics to the state. However, several regional endemics are present in Coahuila, and thus the state serves as a reservoir for regional endemism. In addition, Coahuila is home to several species of conservation concern, especially lizards and turtles. Coahuila thus may be an important state for the conservation of the native regional fauna. Given the relatively unstudied nature of some regions of Coahuila, including the northwestern part of the state that houses two protected areas, the importance of Coahuila may be greater than we currently understand. Indeed, parts of Coahuila have been identified as “species richness hotspots” for lizards (Barrows et al. 2013). In addition, as with the relatively few endemic species, the relative number of species listed as being of conservation concern (i.e., endangered, near threatened, or vulnerable) is also low (22 total in these categories out of 132 native species; 16.7%). We therefore encourage more surveys and more studies on the conservation statuses of the state’s herpetofauna, especially the regions that are now becoming more accessible. This is especially important because as these regions become more accessible to herpetologists, they are also likely to become more susceptible to anthropogenic impacts which could affect the flora and fauna.

Coahuila shares several species with the neighboring states, with the greatest overlap with Nuevo León and Texas. In an analysis of the herpetofauna of the border states of the United States and Mexico, Coahuila frequently clustered with Nuevo León, but was less related to Texas (Smith and Lemos-Espinal 2015). Such overlap is not unexpected, especially given the shared habitats among these states. In particular, the sharing of habitats is likely to be important in explaining the overlap in species composition among states. Indeed, in a comparison of herpetofaunas among the United States-Mexico border states, Smith and Lemos-Espinal (2015) found that the sharing of herpetofaunas paralleled sharing of habitat types. For example, Coahuila shares much of its habitat types with Nuevo León and Tamaulipas, and to a lesser extent with Texas (Smith and Lemos-Espinal 2015). The patterns of shared species are also likely attributed in part to the geological history of the region (Riddle and Hafner 2006).
